# Evaluation Method of Rationality of Creative Genius Cultivation Pattern Based on a BP Neural Network

**DOI:** 10.1155/2022/4639308

**Published:** 2022-09-20

**Authors:** Yong Li, Kailing Dong, Qingyuan Wang

**Affiliations:** ^1^Chengdu University, Chengdu, Sichuan 610106, China; ^2^Chengdu Vocational & Technical College of Industry, Sichuan 610213, China

## Abstract

In order to realize the evaluation of the rationality of an innovative talent training mode, an evaluation model of the rationality of the innovative talent training mode based on a BP neural network is proposed. On the basis of clarifying the principle of the BP neural network model, according to the method of parameter multisource control analysis, the multi-objective characteristic analysis of the rationality of the innovative talent training mode is realized to measure the difference and balance between the two variables of the rationality of the innovative talent training mode. A BP neural network algorithm is designed to find the best feature subset, and a feature sequence sampling and recombination model is constructed. Through reliability evaluation and nonparametric quantitative feature estimation, quantitative analysis and evaluation of the rationality of the innovative talent training mode are realized. The test results show that the confidence level and reliability of the rationality evaluation of the innovative talent training mode using this method are high.

## 1. Introduction

At present, there is a common problem in talent training in colleges and universities to varying degrees: that is, the problem of “short” and “lack” of elements, such as the talent training mode, follows the old system, and the training objectives and specifications of innovation and entrepreneurship are not clear; or there are various problems in the training process, or the management and evaluation system is imperfect or even lacking; or the way and method are single and lack of incentive mechanism. The limitations of its innovation and entrepreneurship education are as follows: only relying on a small number of lectures on entrepreneurship, relying on entrepreneurship plan competitions, or opening a very limited number of entrepreneurship public elective courses, and there is no systematic framework for the entrepreneurship education curriculum system [1–3].

In the traditional methods, the evaluation algorithms for the rationality of creative genius cultivation pattern mainly include the particle swarm optimization (PSO)-based rationality evaluation algorithm, statistical analysis algorithm, and association rule feature extraction algorithm. Through the statistical analysis of the rationality time series of creative genius cultivation pattern [4–6], the rationality evaluation of creative genius cultivation pattern is realized by the method of correlation feature detection. When the adaptability of the above methods for evaluating the rationality of creative genius cultivation pattern is not good, it is necessary to conduct comprehensive and objective analysis and research on the rationality of creative genius cultivation pattern. As an important research method, computational intelligence and knowledge network pay attention to the network relationship between computational intelligence and network structure [7, 8]. Based on this, this paper attempts to effectively combine the knowledge network with the scale to test the rationality of the innovative talent training mode, and try to find the relationship between the dimensions in the scale in order to better evaluate the innovative talent training mode and solve the problem of the innovative talent training [9].

Therefore, this paper puts forward the rationality evaluation model of innovative talent training mode based on a BP neural network. The BP neural network algorithm [10] is used for finding the best feature subset. Based on the feature selection method of regular term, a feature sequence sampling and recombination model for the rationality of the innovative talent training mode is constructed. Combined with the BP neural network and the machine learning method, the reliability evaluation and nonparametric quantitative feature estimation of the rationality of the innovative talent training mode are carried out. The sample regression analysis model is constructed to conduct quantitative analysis and evaluation of the rationality of the innovative talent training mode. Finally, the empirical analysis is carried out and the conclusion of effectiveness is drawn.

## 2. Theoretical Basis of Rationality Evaluation of the Innovative Talent Training Mode

**Figure 1:**
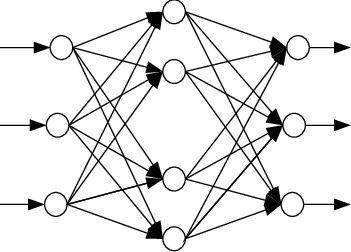
Structure of the BP neural network.

In order to realize the rationality evaluation of the innovative talent training mode based on the BP neural network, it is necessary to first build a statistical analysis model for the rationality evaluation of the innovative talent training mode. The BP neural network is a multilayer feedforward network trained according to error backpropagation (error backpropagation for short). Its algorithm is called the BP algorithm. Its basic idea is the gradient descent method, which uses gradient search technology to minimize the error mean square deviation of the actual output value and expected output value of the network [11, 12].  Figure 1 shows the structure of the BP neural network.

Suppose *x*_*i*_, *y*_*i*_, and *z*_*i*_ are the input layer node, hidden layer node, and output layer node of the BP neural network, respectively. The connection weight between *x*_*i*_ and *y*_*i*_ is *w*_*i*__*j*_; the weight between *y*_*i*_ and *z*_*i*_ is *v*_*i*__*j*_. The detailed learning process is as follows:

1. *Network initialization*. The random value of [−1,1] interval is defined as the network weight and threshold.
2. Output calculation of each layer.
3. Calculate the error of the output layer node.
4. If the error results meet the expected requirements, this is the end. If not, further correction is required.
5. Modify the network weight.
6. Update the network threshold.

**Figure 2:**
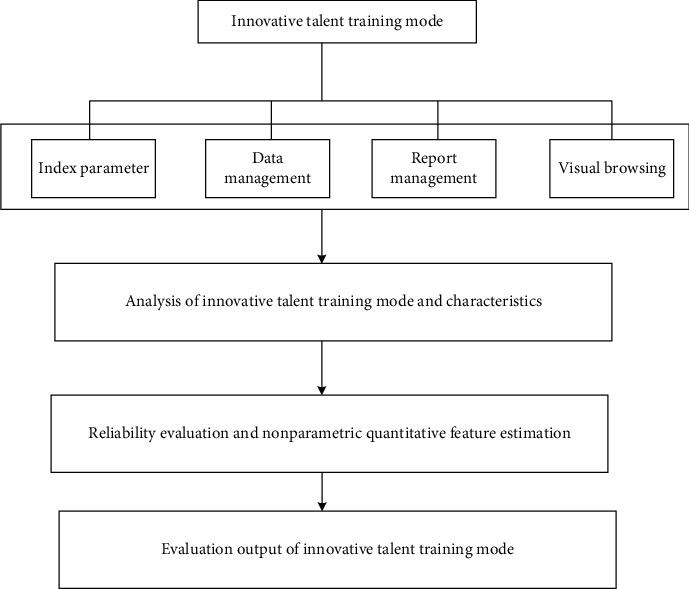
Implementation structure diagram of rationality evaluation of creative genius cultivation pattern based on the BP neural network.

If the calculation result is not within the predetermined range, the above process must be repeated until the output error meets the predetermined interval, and the BP neural network training is ended. Thus, the implementation structure of rationality evaluation of the innovative talent training mode based on the BP neural network is shown in Figure 2.

The structure shown in Figure 2 can be summarized into four parts: (1) the data acquisition part of indicator parameters, data management, report management, and visual management; (2) extraction of the training characteristics of innovative talents; (3) rationality evaluation and nonparametric quantity feature estimation; and (4) output of the evaluation results of the innovative talent training mode.

It is necessary to optimize the curriculum structure of creative genius under the background of talent strategy, emphasize the cultivation of methods and abilities, ensure the education quality of creative genius under the background of talent strategy, and promote the teaching reform of creative genius under the background of talent strategy [13–15].

## 3. Rationality Evaluation Pattern of Creative Talent Cultivation Pattern

### 3.1. Feature Selection of Rationality Evaluation of Creative Talent Cultivation Pattern

Using traditional statistical methods, the nine dimensions in SCL are divided into four categories, and a statistical analysis model for evaluating the rationality of the innovative talent training mode is established. A figure is calculated by some mathematical method. The calculation methods can be divided into three types: (1) the method of calculating based on the known number or quantity—the calculation method follows a specific relationship; (2) the method of calculating by theoretical reasoning of numbers; and (3) the method of calculating based on the average. In this paper, the second one is selected when constructing the statistical analysis model for the rationality evaluation of the new talent training mode.

According to the Shapiro–Wilk (*n* ≤ 50) normality test and variance homogeneity test, we except that the leadership and interpersonal/communication ability dimensions do not meet the normal distribution; the other dimensions meet the normal distribution, and the variance is homogeneous. Therefore, the Mann–Whitney *U* test in the non parametric test is adopted, and the independent-sample *t* test is adopted for the rest. The scores of each dimension of the two groups' self-assessment of talents' ability are inconsistent from high to low. The scores of the two groups' interpersonal/communication ability are the highest, and the scores of the teaching/cooperation ability are the lowest. The details are shown in Table 1.

This paper combines the BP neural network with the machine learning method [16–18]. The reliability evaluation and nonparametric quantitative characteristics of the rationality of the innovative talent training mode are estimated. The correlation gradient function of the rationality of initial creative genius cultivation pattern is expressed as shown in the following formula:

(1) $$\begin{array}{c} x_{ij}=x_{\min\;,j}+\text{rand}\left(0,1\right)\left(x_{\max\;,j}-x_{\min\;,j}\right)\text{,} \end{array}$$

where *x*_min ,*j*_ and *x*_max ,*j*_ represent the minimum and maximum interclass distribution feature sets of the rationality distribution of creative genius cultivation pattern, and rand represents random function. According to the mining results of the rationality evaluation information of creative genius cultivation pattern, frequent item sets are reorganized on the rationality evaluation data of creative genius cultivation pattern [19–21], and the statistical analysis pattern of rationality evaluation of creative genius cultivation pattern is constructed by combining quantitative regression analysis method, and the weighted coefficient is expressed as shown in the following formula:

(2) $$\begin{array}{c} {\left[\nabla^{2}F\left(x\right)\right]}_{kj}≅2J^{T}\left(x\right)J\left(x\right)\text{,} \end{array}$$

where *J*(*x*) and *J*^*T*^(*x*), respectively, represent the dynamic detection factor of the rationality distribution factor of creative talent cultivation pattern.

According to the prior data of the rationality results of creative genius cultivation pattern, a multidimensional distributed task set of the rationality of creative genius cultivation pattern is established as *P*(*n*_*i*_)={*p*_*k*_*|pr*_*kj*_=1, *k*=1,2, ⋯, *m*}, and the fuzzy iterative equation for evaluating the rationality of creative genius cultivation pattern is obtained by the method of priority attribute scheduling, as shown in the following formula:

(3) $$\begin{array}{c} V_{id}^{t+1}=wV_{id}^{t}+c_{1}r_{1}\left(p_{id}-x_{id}\right)+c_{2}r_{2}\left(p_{gd}-x_{gd}\right)\text{,} \end{array}$$

where *w* is the characteristic factor of tree pattern, *c*_1_ and *c*_2_ are dynamic factors for rationality evaluation of creative genius cultivation pattern, *x*_*gd*_ and *x*_*id*_ are dynamic parameters of differential selection, *p*_*gd*_ and *p*_*id*_ are clustering parameters of various factors, and hierarchical clustering parameters are used to determine the correlation characteristic quantity of rationality of creative genius cultivation pattern. For each *w* ∈ *Z*, a statistical analysis and parameter evaluation pattern of rationality of creative genius cultivation pattern are constructed, and the rationality evaluation of creative genius cultivation pattern is carried out by combining the method of big data mining. The objective function of rationality evaluation of creative genius cultivation pattern is as shown in the following formula:

(4) $$\begin{array}{c} F=\overset{n}{\underset{j=1}{\sum }}\overset{m}{\underset{i=1}{\sum }}C_{ij}X_{ij}\text{,} \end{array}$$

where *X*_*ij*_ is the correlation parameter to explain the original variables, *C*_*ij*_ is the factor and principal component characteristics, and *m*, *n* is the hierarchical structure parameter. The analytic hierarchy process and fuzzy parameter fusion method are used to pattern the rationality evaluation of creative genius cultivation pattern, and the characteristic resolution function of rationality evaluation of creative genius cultivation pattern is obtained, as shown in the following formula:

(5) $$\begin{array}{c} g_{k}+A_{k}\Delta x_{k}=0\text{,} \end{array}$$

where *g*_*k*_ is the statistical characteristic quantity of the common factor of the rationality evaluation of creative genius cultivation pattern, *A*_*k*_ is the correlation parameter between the original variables explained by the rationality evaluation factor of creative genius cultivation pattern, and Δ*x*_*k*_ is the comprehensive variable of the rationality evaluation of creative genius cultivation pattern [22]. The statistical big data analysis pattern is established to evaluate the rationality of creative genius cultivation pattern, and the output statistical characteristic quantity is shown in the following formula:

(6) $$\begin{array}{c} \left\{\begin{array}{c} net_{s1}\left(k\right)=r_{s}\left(k\right)\text{,}\\ net_{s2}\left(k\right)=y_{s}\left(k\right)\text{,} \end{array}\right. \end{array}$$

where *r*_*s*_(*k*) represents the original variable of factor interpretation, and *y*_*s*_(*k*) represents the characteristic quantity of principal component. Combining the BP neural network and the machine learning method, the reliability evaluation and nonparametric quantitative characteristic estimation of the rationality of creative genius cultivation pattern are carried out, and the output statistical characteristic quantity is as shown in the following formula:

(7) $$\begin{array}{c} u_{si}\left(k\right)=net_{si}\left(k\right)\text{,} \end{array}$$

where *net*_*si*_(*k*) represents the constraint parameters related to principal components. According to the attribute clustering of big data on the rationality of creative genius cultivation pattern, a multi-objective programming is carried out, and the multi-objective programming function is obtained as shown in the following formula:

(8) $$\begin{array}{c} x_{si}\left(k\right)=\left\{\begin{array}{c} 1,u_{si}\left(k\right)>1,\\ u_{si}\left(k\right),-1\leq u_{si}\left(k\right)\leq 1,\\ -1,u_{si}\left(k\right)<-1, \end{array}\right. \end{array}$$

where *u*_*si*_(*k*) represents the edge node vector of the rational flow load of creative genius cultivation pattern. According to the above analysis, the characteristic sequence sampling and recombination pattern of creative genius cultivation pattern rationality is constructed, and the reliability evaluation of creative genius cultivation pattern rationality is carried out by combining the BP neural network and the machine learning method [23–25].

The entity load of rationality of creative genius cultivation pattern is as follows: fuzzy evaluation and parameter evaluation of rationality of creative genius cultivation pattern are carried out using the method of association regularity detection, and the output migration load of creative genius cultivation pattern is obtained as shown in the following formula:

(9) $$\begin{array}{c} L_{t}^{eff}=\frac{1}{n_{j}}\frac{L_{0}P_{j}^{\min}-L_{j}P_{0}^{\min}}{P_{0}^{\min}+P_{j}^{\min}}\text{.} \end{array}$$

In the above formula, *P*_*j*_^min^ represents the modified optimal load transfer of the rationality of creative genius cultivation pattern, *P*_0_^min^ represents the decision variable of the rationality evaluation of creative genius cultivation pattern, *X*_*ij*_ is the autocorrelation variable of the rationality evaluation of creative genius cultivation pattern, and *n*_*j*_ represents the marginal feature distribution of the rationality evaluation of creative genius cultivation pattern, thus realizing the feature screening of the rationality evaluation of creative genius cultivation pattern.

### 3.2. Output of Rationality Evaluation of Creative Talent Cultivation Pattern

The characteristic sequence sampling and recombination pattern of the rationality of creative genius cultivation pattern is constructed. Combining the BP neural network and the machine learning method [26], the reliability evaluation and nonparametric quantitative characteristic estimation of the rationality of creative genius cultivation pattern are carried out. The optimization control module of the rationality evaluation of creative genius cultivation pattern is described as shown in the following formula:

(10) $$\begin{array}{c} \min\left(f\right)=\overset{m}{\underset{i=1}{\sum }}\overset{n}{\underset{j=1}{\sum }}C_{ij}X_{ij},\\ =\left\{\begin{array}{c} \overset{m}{\underset{j=1}{\sum }}X_{ij}=a_{i},i=1,2\cdots m\text{,}\\ \overset{m}{\underset{i=1}{\sum }}X_{ij}=b_{i},j=1,2\cdots n\text{,}\\ X_{ij}\geq 0,i=1,2\cdots m,j=1,2\cdots n\text{,} \end{array}\right. \end{array}$$

where *C*_*ij*_ and *X*_*ij*_ are related. Through the above mathematical pattern construction, a multi-objective programming pattern for evaluating the rationality of creative genius cultivation pattern is established, which is expressed as shown in the following formula:

(11) $$\begin{array}{c} x_{i}=\left\{\begin{array}{c} 0,M-\overset{\lfloor n/2\;}{\underset{j=1}{\sum }}w_{j}-\overset{i}{\underset{j=\lfloor n/2\;+1}{\sum }}w_{j}-\overset{k}{\underset{j=i+1}{\sum }}w_{j}<0,i\leq k\text{,}\\ f_{i}\left(M,n,w,c,r\right)=\min\;\left\{f\left(M,n,w,c,r\right)\right\}\text{,}\\ 1,M-\overset{\lfloor n/2\;}{\underset{j=1}{\sum }}w_{j}-\overset{i}{\underset{j=\lfloor n/2\;+1}{\sum }}w_{j}-\overset{k}{\underset{j=i+1}{\sum }}w_{j}>0\text{,} \end{array}\right. \end{array}$$

where *M* is the original variable of factor explanation of creative genius cultivation pattern, *w*_*j*_ is the statistical characteristic quantity of common factor, and *f*_*i*_(*M*, *n*, *w*, *c*, *r*) is the positive correlation parameter between factors. Through the autocorrelation feature matching method, intelligent learning and output control can be achieved, and the statistical characteristic quantity of rationality evaluation of creative genius cultivation pattern meets the multi-objective linear mapping. The mapping relationship between multi-objective programming constraint parameter set *R*^*N*^ and *X*^*N*^ existing correlation is shown in the following formula:

(12) $$\begin{array}{c} p\left(R^{N}=r_{i}\right)=p\left(\begin{array}{c} \left.X^{N}=x_{i}\right|\left|x_{i}\right|=\left|r_{i}\right|,angle\left(x_{i}\right)\\ =\left(angle\left(r_{i}\right)-\varphi_{g}\right)\mathrm{mod}\;\left(2\pi \right) \end{array}\right)\text{,} \end{array}$$

where *X*^*N*^ is the total score parameter of creative genius cultivation pattern, *x*_*i*_ is the correlation parameter of creative genius cultivation pattern, *r*_*i*_ is the dynamic distribution parameter of creative genius cultivation pattern, and *angle*(*x*_*i*_) is the multivariate statistical characteristic parameter of creative genius cultivation pattern. Combining with the artificial intelligence learning method, the self-adaptive learning of rationality evaluation of creative genius cultivation pattern is obtained, and the reliability constraint parameter pattern describing rationality evaluation of creative genius cultivation pattern is as shown in the following formula:

(13) $$\begin{array}{c} H\left(R^{N}\right)=-\overset{M}{\underset{i=1}{\sum }}p\left(r_{i}\right)lg\left(p\left(r_{i}\right)\right)\text{,} \end{array}$$

where *M* is the number of elements in the symbol set, the fuzzy characteristic analysis method is adopted, *p*(*r*_*i*_) is the correlation parameter between the original variables in the rationality evaluation of creative genius cultivation pattern, *p*(*x*_*i*_) is the explanation process parameter of the rationality factor of creative genius cultivation pattern, and *X*^*N*^ is the fuzzy characteristic quantity of the rationality of creative genius cultivation pattern, so as to evaluate the rationality of creative genius cultivation pattern.

**Figure 3:**
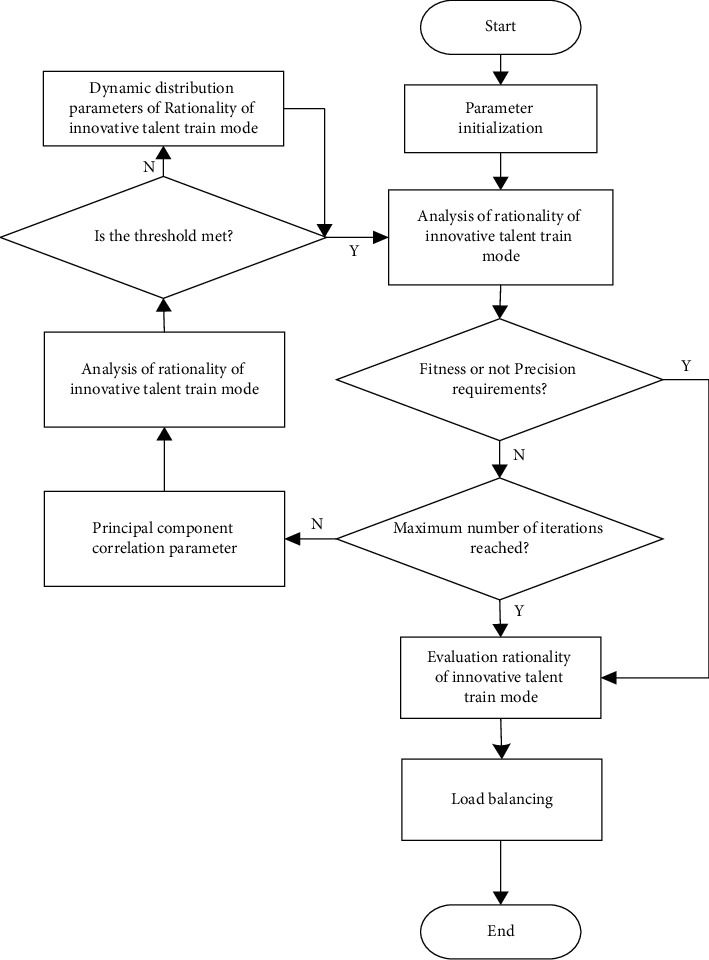
Optimization process of rationality evaluation of creative talent cultivation pattern.

Combining the BP neural network with the machine learning method, the reliability evaluation and nonparametric quantitative feature estimation of the rationality of creative genius cultivation pattern are carried out, and the sample regression analysis pattern is constructed to quantitatively analyze and evaluate the rationality of creative genius cultivation pattern; the random distribution concept set of the rationality evaluation of creative genius cultivation pattern is constructed, and the random probability distribution is obtained as shown in the following formula:

(14) $$\begin{array}{c} H=H\left(R^{N},\varphi_{g}|Z^{N}\right)=H\left(R^{N}|Z^{N}\right)+H\left(\varphi_{g}|Z^{N}\right)-I\left(R^{N};\varphi_{g}|Z^{N}\right)\text{,} \end{array}$$

where the objective window function of rationality evaluation of creative genius cultivation pattern is *R*_2_^*T*^*R*_2_=*V*_2_∑_2_*V*_2_^*T*^, the fuzzy correlation scheduling of rationality evaluation of creative genius cultivation pattern is carried out by the multidimensional hierarchical mining method, the rationality of creative genius cultivation pattern is statistically evaluated by the rough set evaluation and multi-objective programming method, and the optimal learning weight of rationality evaluation of creative genius cultivation pattern is obtained as shown in the following formula:

(15) $$\begin{array}{c} \omega =\omega_{\max}-t\frac{\omega_{\max}-\omega_{\min}}{T_{\max}}\text{,} \end{array}$$

where *ω*_max_ and *ω*_min_, respectively, represent the regulation coefficient of the rationality evaluation of creative genius cultivation pattern, and *T*_max_ is the maximum control timescale. Based on the above analysis, a characteristic sequence sampling and recombination pattern of the rationality of creative genius cultivation pattern is constructed. Combining the BP neural network and the machine learning method, the reliability evaluation and nonparametric quantitative feature estimation of the rationality of creative genius cultivation pattern are carried out, and a sample regression analysis pattern is constructed to quantitatively analyze and evaluate the rationality of creative genius cultivation pattern, so as to realize the rationality evaluation of creative genius cultivation pattern. The optimization process is shown in Figure 3.

According to the above optimization process, the rationality evaluation and optimization of the innovative talent training mode are realized. The optimized evaluation model has better evaluation accuracy and efficiency.

## 4. Simulation Analysis

**Figure 4:**
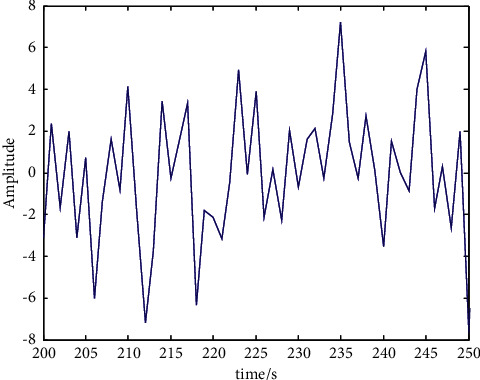
Cluster ice chart of each factor in the rationality evaluation of creative talent cultivation pattern.

**Figure 5:**
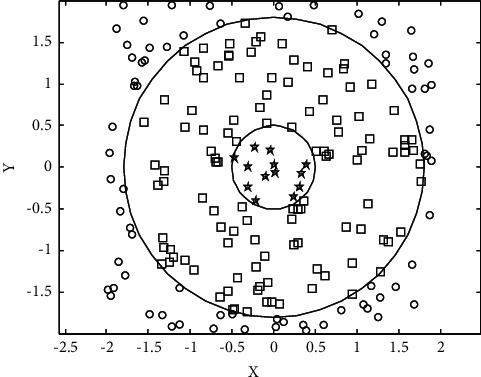
Cluster tree diagram of each factor in the rationality evaluation of creative talent cultivation pattern.

In order to verify the application performance of this method in evaluating the rationality of creative genius cultivation pattern, a simulation test is conducted, and a simulation analysis is conducted by combining 14.0 Matlab 7 and SPSS14.0. When analyzing the cluster diagram of each factor of SCL-90, hierarchical clustering is adopted, which can also be called systematic clustering. In SPSS, the path is analysis-classification-systematic clustering, and variables are selected in clustering. The cluster ice wall chart and cluster tree chart of each factor are analyzed, as shown in Figures 4 and 5, respectively.

Under the background of talent strategy, the management department of creative genius and 201 creative genius under the background of talent strategy conducted a questionnaire survey (209 valid questionnaires were collected in total). The number of people counted was 2,000, and the number of optimization iterations of multi-objective programming was 400. According to the above simulation parameters, the rationality evaluation simulation analysis of creative genius cultivation pattern was carried out.

**Figure 6:**
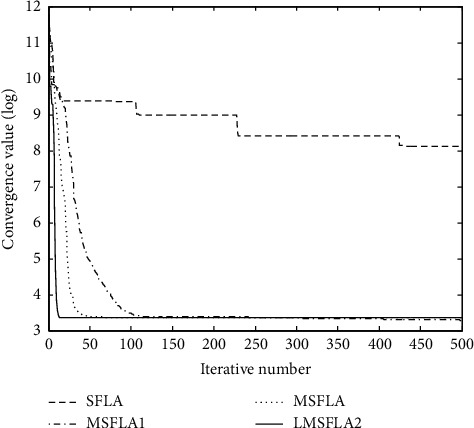
Distribution of features of rationality evaluation of creative talent cultivation pattern.

Taking the data in Figures 3 and 4 as the research object, the fuzzy correlation big data analysis pattern of the rationality of creative genius cultivation pattern is established, and the fuzzy degree evaluation and parameter evaluation of the rationality of creative genius cultivation pattern are carried out by the method of association regularity detection. The characteristic distribution diagram is shown in Figure 6.

Using the factor analysis method, 748 positive test data were screened, and the results of discipline construction, scientific research, teaching, and achievements were transformed into factors. According to the analysis of Figure 5, this method has good convergence when evaluating the training mode of innovative talents.

The test confidence level is shown in Table 2.

According to the analysis, the rationality evaluation of creative genius cultivation pattern by this method has high confidence level and good reliability, and the evaluation results can accurately reflect the subset of evaluation features related to the rationality of creative genius cultivation pattern. The rationality evaluation parameters obtained by the evaluation all have significant differences (*P* < 0.05), while other factors have no significant differences. The rational distribution of creative talent cultivation pattern is lower than the norm (*P* < 0.05), and the talent cultivation is significantly higher than the norm (*P* < 0.05).

**Figure 7:**
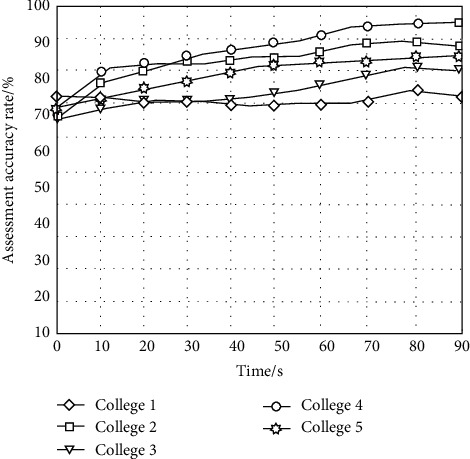
Evaluation accuracy.

In order to further test the reliability of the algorithm in this paper in evaluating the rationality of the innovative talent training mode, statistics are made on the evaluation accuracy of the five universities in the innovative talent training mode using the algorithm in this paper. The statistical results are shown in Figure 7.

It can be seen from the results in Figure 7 that the accuracy rate of evaluating the rationality of the innovative talent training mode using the algorithm in this paper is over 65%, which indicates that the algorithm in this paper can not only effectively evaluate the rationality of the innovative talent training mode, but also has a high evaluation accuracy rate.

**Figure 8:**
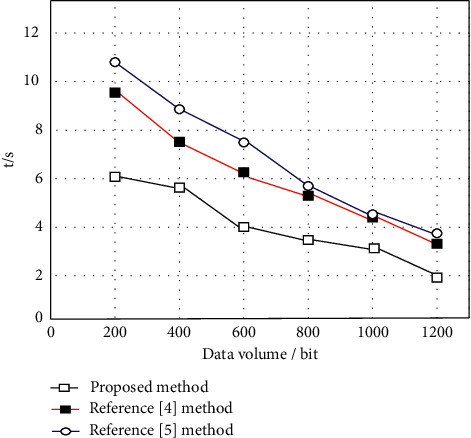
Comparison of evaluation time between different methods.

On this basis, the computational complexity of the proposed algorithm is calculated by comparing the methods in [4, 5]. The shorter the time of the algorithm, the lower the computational complexity. Therefore, the experiment is conducted with the evaluation time as the test index. The experimental results are shown in Figure 8.

As shown in Figure 8, under the same conditions, the time of the proposed method is the shortest and the maximum time is 6S, which indicates that the proposed method has the lowest computational complexity, the strongest operability, and high practical applicability.

## 5. Discussion

### 5.1. Deficiency

1. The cultural atmosphere of innovation needs to be strengthened.
2. Innovation requires a free, relaxed, and failure-tolerant cultural atmosphere and innovation environment. Although many colleges and universities in China are constantly exploring the innovative talent training mode and formulating the corresponding innovative talent training plans and programs, the cultivation of innovative talents in some colleges and universities is still on the surface, and innovation is not really implemented in teaching.
3. Lack of innovative teachers.
4. At present, there is a lack of innovative and double-qualified teachers in colleges and universities in China. Although some college teachers have high academic qualifications, they lack corresponding work experience, social practice, and innovation consciousness. Teachers in colleges and universities still focus on theoretical guidance and adopt a single teacher-based teaching method. They fail to combine theoretical knowledge with social practice in the teaching process, and cannot actively guide students to develop innovative thinking, innovative spirit, and innovative ability. In addition, some teachers are busy with their own scientific research, pay little attention to the cultivation of students' innovative abilities, and neglect the development of students' innovative potential. The lack of innovative and double-qualified teachers in colleges and universities is far from meeting the needs for cultivating innovative talents in colleges and universities.
5. The curriculum system of innovative talents training is not perfect.
6. The imperfection of the curriculum system in colleges and universities is mainly reflected in two aspects: first, undergraduate education lacks courses aimed at cultivating innovative talents. Higher education in China is divided into vocational education and theoretical education. For a long time, higher vocational colleges mainly undertake the task of vocational education, while undergraduate schools mainly undertake theoretical education. Therefore, many undergraduate schools have more theoretical courses, but less or no practical courses for the cultivation of innovative talents; and second, the curriculum system for the cultivation of innovative talents is unreasonable. At present, the practical courses for the cultivation of innovative talents in many colleges and universities are only case studies in the classroom, which do not allow students to really participate in innovative activities, and fail to enable students to think actively when solving practical problems with theoretical knowledge and cultivate innovative ability. Such a curriculum system is still not separated from theoretical teaching.
7. The evaluation mechanism of the innovative talent training is not perfect.
8. At present, the evaluation mechanism of university teachers' talent training does not include the innovative ability in the assessment criteria, which leads to the teachers blindly pursuing the publication of papers, books, and scientific research projects. Therefore, it is difficult to form a long-term innovative cultural atmosphere, and the quality of the innovative talent training cannot be improved.

### 5.2. Strategy

1. We should improve classroom teaching efficiency and lay a solid foundation for students' innovation.
2. We are in the era of knowledge economy and information technology. Compared with their predecessors, today's students need to learn so much and have greater pressure. Therefore, we must teach students the most basic and applicable things, teach them how to learn, how to do things, and how to be a person, and encourage them to be brave in challenges and good at entrepreneurship.
3. We should reasonably set up practice links to improve students' innovation ability.
4. attach importance to teaching practice and highlight the creative nature of education. It is an effective way to cultivate students' practical ability and arouse their enthusiasm for innovation to emphasize the link to practical teaching and attach importance to the combination of theory and practice. Social work and social practice play a special role in improving students' comprehensive quality, and cultivating students' practical ability and innovative spirit. According to the requirements of social and economic development, the university pays attention to updating the practice teaching links and contents, advocates the integration of practice activities with production practice, and strengthens the construction of practice. We should make full use of social resources, make students participate in enterprise production and operation activities during school, shorten the distance between school and society, and improve students' practical ability.
5. We should closely rely on scientific research and training to cultivate students' innovative consciousness.
6. We should study how to vigorously develop the third class of industry university research cooperation, and adopt various ways to attract students with certain innovation ability to participate in the scientific research work of teachers so that students in school can gain knowledge in practice, increase wisdom in scientific research, and further expand the ways of using educational resources to train application-oriented talents.
7. We should actively participate in competition activities and induce students' innovative thinking.
8. The competition is a banner leading modern college students to ignite their passion for innovation, and an important carrier to promote the cultivation of innovative talents in colleges and universities and the construction of an innovative country. Organizing competitions can not only inspire young students' fighting spirit, arouse their innovative consciousness, and cultivate their innovative spirit and ability, but also promote the creation of a positive campus cultural atmosphere and the activation of students' extracurricular scientific and technological activities.
9. We should carry out the second class extensively and expand students' innovation space.
10. The second class is an important part of talent training, an important way for students to practice professional theoretical knowledge and cultivate innovative and practical abilities, and an effective means to improve students' employment competitiveness. Through practice and exploration, make full use of the form of students' professional associations, actively organize students to participate in various social practice activities, and gradually form a set of second class implementation plans that are suitable for the training objectives of the major, meet the teaching requirements, and have a relatively perfect system.

## 6. Conclusions

In this paper, the rationality evaluation model of the innovative talent training mode based on the BP neural network is proposed. A statistical analysis model is built to evaluate the rationality of the innovative talent training mode, and the multi-objective planning and characteristics of the rationality of the innovative talent training mode are analyzed. The fuzzy evaluation and parameter evaluation are carried out using the method of association rule detection to realize the rationality evaluation of the innovative talent training mode. The empirical analysis results show that this method has a high accuracy in evaluating the rationality of creative genius cultivation pattern, and it has a good application value in evaluating and predicting the rationality of creative genius cultivation pattern.

## Figures and Tables

**Table 1:** Ranking of the average scores of each dimension of the self-assessment of the two groups of students.

Project	Sort/rank	
	Experimental group (36 students)	Control group (40 students)
Leadership	5	4
Interpersonal/communication skills	8	7
Teaching/cooperation ability	11	10
Professional development ability	6	5

**Table 2:** Confidence level test of rationality evaluation of creative talent cultivation pattern.

Test object	Subject	Scientific research	Teaching	Achievement transformation
1	6.914	4.645	76.822	0.574
2	8.164	6.039	43.630	3.956
3	6.834	7.057	42.142	7.798
4	1.755	8.355	34.345	8.470
5	0.826	3.852	58.740	6.270
6	9.928	3.331	91.603	1.912
7	7.342	5.362	77.898	9.333
8	2.739	9.961	13.184	6.011
9	9.794	0.465	67.191	5.985
10	5.407	9.642	32.633	5.679
11	5.505	0.929	12.637	8.074
12	6.771	6.357	77.919	2.099
13	2.308	9.730	66.212	2.991
14	9.493	0.694	81.183	3.316
15	2.804	3.972	14.461	9.432
16	1.151	8.263	12.349	4.159
17	0.663	3.854	58.293	3.454
18	0.254	8.942	3.119	4.646
19	413.211	9.259	96.540	9.152
20	409.572	5.291	20.348	8.138

## Data Availability

The raw data supporting the conclusions of this article can be obtained from the corresponding author upon request.
